# Cardiovascular Screening for the Asymptomatic Patient with Diabetes: More Cons Than Pros

**DOI:** 10.1155/2017/8927473

**Published:** 2017-12-14

**Authors:** Konstantinos Makrilakis, Stavros Liatis

**Affiliations:** First Department of Propaedeutic Internal Medicine, National and Kapodistrian University of Athens Medical School, Laiko General Hospital, Athens, Greece

## Abstract

Diabetes mellitus is associated with an increased risk of coronary heart disease (CHD) morbidity and mortality. Although it frequently coexists with other cardiovascular disease (CVD) risk factors, it confers an increased risk for CVD events on its own. Coronary atherosclerosis is generally more aggressive and widespread in people with diabetes (PWD) and is frequently asymptomatic. Screening for silent myocardial ischaemia can be applied in a wide variety of ways. In nearly all asymptomatic PWD, however, the results of screening will generally not change medical therapy, since aggressive preventive measures, such as control of blood pressure and lipids, would have been already indicated, and above all, invasive revascularization procedures (either with percutaneous coronary intervention or coronary artery bypass grafting) have not been shown in randomized clinical trials to confer any benefit on morbidity and mortality. Still, unresolved issues remain regarding the extent of the underlying ischaemia that might affect the risk and the benefit of revascularization (on top of optimal medical therapy) in ameliorating this risk in patients with moderate to severe ischaemia. The issues related to the detection of coronary atherosclerosis and ischaemia, as well as the studies related to management of CHD in asymptomatic PWD, will be reviewed here.

## 1. Introduction

The prevalence of diabetes mellitus (DM) is increasing and is reaching epidemic proportions worldwide, as the population becomes older and is less active and more obese. According to recent estimates from the International Diabetes Federation (IDF), approximately 415 million people were affected by DM globally in 2015, with the projections being very dire for the future (642 million people are predicted for the year 2040) [1]. The economic burden of treating diabetes and its complications is likewise enormous [2].

People with diabetes (PWD) are at increased risk of developing both micro- and macrovascular complications [3], which are diminished with proper glycaemic treatment [4]. Compared with people without diabetes, men and, especially, women with diabetes have decreased life expectancy (six to eight years less) [5]. One needs to keep in mind though that these people do not die from diabetes per se but rather from cardiovascular disease (CVD) [6, 7]. At the time of diagnosis of type 2 diabetes (T2D), many patients already have one or more additional risk factors for macrovascular disease (obesity, hypertension, dyslipidaemia, and smoking) and many have evidence of overt atherosclerosis (previous myocardial infarction (MI), ischaemic stroke, ischaemic changes on electrocardiogram (ECG), or peripheral vascular disease) [8].

The relationship linking DM to CVD is however more complex and multifaceted in nature [9]. Apart from the above-mentioned classical CVD risk factors, studies have reported that several other factors, including increased oxidative stress, increased coagulability, low-grade inflammation, endothelial dysfunction, and autonomic neuropathy, are often present in patients with DM and may directly contribute to the development of CVD [10]. It has been shown that endothelium-dependent epicardial coronary artery vasodilation in response to acetylcholine [11] or physiological stimuli [12] is impaired in diabetic patients, suggesting that endothelial dysfunction (known to be an independent predictor of cardiovascular events [13]) occurs before the development of overt atherosclerosis in these persons. Furthermore, nonobstructive coronary atherosclerosis (defined as either <50% luminal narrowing, <20% narrowing, or unimpaired coronary blood flow, depending on the study [14]) is also frequently associated with both DM [15, 16] and vascular dysfunction [17], thus independently contributing to the increased CVD event risk [18]. Collectively, the high rates of CVD risk factors and direct biological effects of diabetes on the cardiovascular system place diabetic persons at very increased risk of developing CVD [9].

Coronary heart disease (CHD) is the leading cause of morbidity and mortality in PWD, as it is implicated in 60%–80% of deaths and is 2–4 times more common in the diabetic compared to the general population [19]. CHD has certain unique characteristics in diabetic persons, making it more serious and aggressive. It is often more extensive, affecting multiple, more peripheral, and smaller blood vessels [20], accompanied by generalized endothelial dysfunction and microcirculatory disorders in the myocardium [21], thus resulting in more difficult revascularization procedures, compared to the nondiabetic individuals [22]. The atherosclerotic plaque of diabetic patients is also infiltrated by macrophages more extensively and has more lipid components, making it more unstable and vulnerable to rupture [23]. Furthermore, CHD in PWD occurs at a younger age (on average 15 years earlier) [24] and has a higher mortality in diabetic women [25]. Also, CHD in diabetes is more often silent (not producing clinical subjective symptoms), which makes it difficult to diagnose early [26, 27]. It is believed that this fact—the nonperception of pain that results in silent ischaemia, atypical symptoms, or even silent myocardial infarctions—is, at least in part, due to autonomic denervation of the heart [28]. Multivessel CHD is also common in asymptomatic patients with diabetes, particularly those with two or more coronary risk factors other than diabetes [29].

Due to all these special characteristics, the prognosis of CHD in diabetic persons is worse than in nondiabetic ones: even after myocardial revascularization (coronary artery bypass grafting (CABG) or percutaneous coronary intervention (PCI) with stent placement), restenosis rates and perioperative or long-term outcomes are worse in diabetic individuals [22, 30, 31]. Thus, it is considered by many that DM is equivalent to established CHD in terms of cardiovascular prognosis, that is, that PWD with no history of CHD has the same risk of developing a cardiovascular event in the future compared to nondiabetic ones who already have CHD manifestations. This statement was actually based on a few studies [32–34], in which T2D patients without history of CHD events at baseline showed similar coronary mortality as nondiabetic patients who had a previous coronary event. On the other hand, recent studies [35] and meta-analyses [36] indicate that a significant part of PWD are in a lower cardiovascular risk category (e.g., men younger than 35 years of age, women younger than 45 years, and patients with diabetes duration of less than 10 years without other risk factors) [37], and recent guidelines do not anymore consider diabetes as a CHD risk equivalent, but recommend cardiovascular risk stratification for primary and secondary prevention [38, 39].

The risk of developing CHD is actually beginning to increase from the prediabetic phase, prior to the clinical manifestation of DM. Many of the atherogenic risk factors are present already in the prediabetic phase, as most individuals have characteristics of the metabolic (insulin resistance) syndrome, which increases CVD risk [40]. The CHD risk in PWD varies widely with the intensity of these risk factors. The evidence is strongest for hypertension, elevated low-density lipoprotein cholesterol, smoking, the metabolic syndrome, hyperglycaemia, and microalbuminuria, and it is generally recommended that all these should be aggressively treated, so that the risk of future CHD events will be decreased [41].

Taking all this information into consideration, there is no doubt that, compared to individuals without diabetes, those with diabetes have a higher prevalence of CHD, a greater extent of coronary ischaemia, and are more likely to suffer a myocardial infarction and silent myocardial ischaemia. Furthermore, the risk of asymptomatic CVD in diabetic persons is quite high [42]. Although there is a preponderance of evidence that in the setting of an acute coronary syndrome an invasive approach using coronary revascularization has a morbidity and mortality benefit [43], the optimal strategy for detection and management of CHD in stable, asymptomatic persons is not very well elucidated [44]. The extent to which routine revascularization reduces death or MI, or improves quality of life (QoL) in patients with stable ischaemic heart disease (SIHD), on top of guideline-directed optimal medical therapy (GDOMT), represents one of the greatest uncertainties in cardiology [45]. Accordingly, although screening to detect early CHD and provide targeted treatment for patients with possible evidence of CHD would seem reasonable, there are multiple issues that must be addressed concerning the possible role of screening in such patients (e.g., the accuracy of screening tests, how easily testing can be performed, the safety and costs of testing, and available interventions that improve outcomes after screening).

In this context, issues related to the detection of coronary atherosclerosis and ischaemia, as well as the studies related to management of CHD in asymptomatic patients with diabetes, will be reviewed here.

## 2. Presymptomatic Screening for CHD in Diabetes

Screening is defined as an attempt to identify an asymptomatic, unrecognized disease before its clinical presentation or risk factor for it. This is done by history taking (e.g., asking about smoking), physical examination (e.g., a blood pressure measurement), laboratory test (e.g., serum cholesterol measurement), or another procedure (e.g., a cervical Pap-test in women) that can be applied to asymptomatic people. It is thus hoped that screening may improve the effectiveness of secondary prevention, which aims at preventing the progression and clinical onset of the disease in people without yet clinical signs, by identifying those who already have the disease without knowing, and who are likely to benefit from the early diagnosis.

In 1971, Archie Cochrane, considered one of the originators of Randomized Clinical Trials and Evidence-Based Medicine, wrote in his groundbreaking paper on validation of medical screening procedures: “If a patient asks a medical practitioner for help, the doctor does the best he can. He is not responsible for defects in medical knowledge. If, however, the practitioner initiates screening procedures, he is in a very different situation. He should have conclusive evidence that screening can alter the natural history of disease in a significant proportion of those screened.” [46].

A unique requirement for screening in secondary prevention is that treatment of early, asymptomatic disease must be superior to treatment of the disease when it would have been diagnosed in the usual course of events, when a patient will seek medical care for symptoms. If the outcome in the two situations is the same, screening does not add value.

Thus, in the case of asymptomatic CHD in PWD, it is not enough just to find early that they suffer from CHD, but also to prove that early, asymptomatic diagnosis will lead to a reduction in cardiac morbidity and/or mortality, by applying some form of intervention, other than the one(s) that is (are) already recommended and should be applied anyway (i.e., control of the CHD risk factors). Given the increased risk of CHD in the case of PWD, GDOMT involves the use of “disease-modifying” pharmacological interventions, including statins, inhibitors of the renin-angiotensin-aldosterone axis, and possibly antiplatelet agents, that individually have been shown to reduce death and MI in placebo-controlled trials, together with lifestyle interventions, such as cigarette smoking cessation, prudent diet, and regular exercise [45]. Control of hyperglycaemia has also been proven to be beneficial, especially if started early, in both types of diabetes [4, 47].

The goal of CHD treatment is to alleviate symptoms of angina (if present) and, most importantly, to protect the patient from subsequent serious complications, that is, MI, heart failure, or even death. Although angina has long been considered the cardinal symptom of myocardial ischaemia and CHD, silent (asymptomatic) myocardial ischaemia is the most common manifestation of CHD, accounting for more than 75% of ischaemic episodes during daily life (both in people with known CHD and in those with unknown disease) [48]. Silent myocardial ischaemia is defined as the presence of objective evidence of myocardial ischaemia in the absence of chest discomfort or another anginal equivalent symptom (e.g., dyspnoea, nausea, and diaphoresis). Objective evidence of silent myocardial ischaemia in PWD may be obtained through several ways [49], and its prevalence depends significantly on the method of screening and what test result is considered diagnostic for CHD. The available screening tests can be divided into invasive (e.g., coronary artery angiography) or noninvasive and functional or anatomic ones [50] (Table 1). Since catheter-based coronary artery angiography [51] is expensive and is associated with a small risk of serious complications that are directly related to its invasive nature and to the use of iodinated contrast media (such as atheroembolism, bleeding, myocardial infarction, ventricular tachyarrhythmias, renal failure, stroke, and death), noninvasive techniques, are usually preferred first and gaining great popularity. Furthermore, coronary angiography is also hampered by technical limitations, such as the occasional inability to optimally visualize a particular location and also by providing information only about the contour of the vascular lumen and not the components of the vascular wall, which are not visualized. Atherosclerotic plaques develop initially in the vascular wall and can thus be missed or their significance underestimated by coronary lumen angiography, which is not generally recommended for screening purposes [52].

The following tests are available for the noninvasive diagnosis of CHD:

- Exercise ECG, generally using a treadmill and standardized protocols [53–55]
- Radionuclide myocardial perfusion imaging (rMPI) using either exercise or pharmacologic (dobutamine, adenosine, or dipyridamole) stress and imaging with either single photon emission computed tomography (SPECT) [27, 56] or positron emission tomography (PET) [57]
- Echocardiography using either exercise or pharmacologic stress [58, 59]
- Cardiac magnetic resonance (CMR) imaging [60]
- Coronary computed tomography, either for detection of coronary artery calcifications (CAC) or for assessing coronary artery stenosis with coronary computed tomography angiography (CCTA) [61]
- Hybrid imaging, using either SPECT/CT, PET/CT, or PET/MRI [62]

Stress testing provides physiologic evidence of clinically significant coronary artery stenoses by demonstrating the effects of diminished coronary flow reserve on symptoms, characteristic changes on the ECG, myocardial perfusion defects on scintigraphy or PET, or regional wall motion abnormalities on echocardiography. Nondiagnostic findings or ambiguity on the results may suggest the need for direct (anatomic) assessment of the coronary artery lumen with invasive (or noninvasive) coronary arteriography.

Stress testing (either with exercise or pharmacologic provocation) combined with radionuclide myocardial perfusion imaging (rMPI) is more sensitive than exercise stress testing alone (despite the latter's simplicity, wide availability, and low cost) for the diagnosis of CHD, in both people with and without diabetes [63]. rMPI is particularly appealing for screening asymptomatic PWD [64] and has a central role in the diagnosis and risk stratification of diabetic patients with suspected CHD, in particular for the evaluation of silent ischaemia [56, 65]. The reported prevalence of silent myocardial ischaemia on rMPI in DM patients has been disparate among studies [49]. Observational studies performed more than a decade ago reported a prevalence ranging from 16% to 59%, with approximately 20% of patients having high-risk findings [27, 66], whereas in more recent studies, a much lower prevalence of any perfusion defect or LV function abnormality (22% in DIAD (Detection of Ischemia in Asymptomatic Diabetes) study) [42] or even lower (12.5%) [67] has been reported. The yield of stress testing in asymptomatic PWDM can be improved by selecting patients based on the pretest clinical risk of CHD, for example, by selecting persons with abnormal ECGs and vascular disease [66] or—if performed—with a high CAC score (a CAC score > 400 or >1000 is predictive of moderate to severe silent myocardial ischaemia on SPECT in diabetic patients (48% and 71% ischaemia, resp.)) [68]. Limitations of the technique include the fact that it is expensive and diagnostic image quality is affected in obese patients, as well as in women and men with large breasts. Also, global reductions in myocardial perfusion, such as in the setting of left main or 3-vessel CHD, can result in balanced reduction and an underestimation of ischaemic burden with myocardial perfusion SPECT.

Several other imaging variables with high diagnostic and prognostic value can also be obtained during SPECT-MPI. Among them, transient ischemic dilation (TID) [69, 70], defined as the apparent presence of left ventricular dilation on poststress relative to rest images [71], has been linked to increased CVD risk in the context of reversible myocardial perfusion defects during SPECT-MPI [72, 73]. Specifically for diabetic persons, TID provides independent and incremental prognostic information for the prediction of cardiac death or nonfatal MI [74], and even in the absence of regional myocardial perfusion abnormalities, TID is an important sign of CHD, especially when TID ratio exceeds 1.16 [75].

Furthermore, adverse remodeling of the left ventricle (LV), defined as a change in shape due to CVD, is associated with worse prognosis [76], as shown, for example, with the increased end-systolic volume in patients after myocardial infarction [77]. Left ventricular geometry is especially associated with the pathophysiology and symptomatology of congestive heart failure (CHF) [78], usually assessed with echocardiography [79]. Of note, incidental diagnosis of left ventricular systolic dysfunction (LVD) is common in clinical practice. The prevalence of asymptomatic LVD (ejection fraction (EF) < 50%) is 6.0% in men and 0.8% in women of the general population and is twice as common as symptomatic LVD. The timely and definitive exclusion of an ischaemic etiology is central to optimizing care and reducing mortality in this case. Advances in cardiovascular imaging provide many options for imaging of patients with LVD [80]. Gated myocardial perfusion single photon emission computed tomography (SPECT) (MPS) has the ability to provide operator-independent measurements of myocardial perfusion and function in 3 dimensions [81], thus providing more precise information regarding the LV shape [82]. The left ventricular shape index (LVSI), derived as the ratio of maximum 3D short- and long-axis LV dimensions, for end systole and end diastole, has been shown to be an independent predictor of CHF hospitalization [81] and can potentially be used for the detection of ischemia even in the absence of a perfusion defect in the territory of a specific coronary artery [83].

Positron emission tomography (PET) has several clinical and research applications in cardiovascular imaging but is still not widely utilized in routine clinical practice because of nonuniversal availability of PET scanners (cardiac PET tracers are costly and require either an onsite cyclotron or a monthly generator). PET/CT hybrid cameras are superior to PET, SPECT/CT, and SPECT scanners. PET provides better image quality because of higher spatial resolution, less scatter, and fewer attenuation artifacts. ^18^F-FDG PET imaging has high sensitivity for the detection of hibernating/viable myocardium and has replaced Tl-201 SPECT imaging in centers equipped with a PET/CT camera [62]. Myocardial perfusion imaging with PET allows accurate global and regional measurements of myocardial perfusion, myocardial blood flow, and function at stress and rest in a single study session performed in approximately 30 min [57, 84]. The noninvasive assessment of coronary flow reserve (CFR = stress divided by rest myocardial blood flow) using PET is a powerful tool that integrates the effects of focal stenosis, diffuse disease, and coronary microvascular function and has been shown that impaired CFR (below the median) was associated with an adjusted 3.2- and 4.9-fold increase in the rate of cardiac death for diabetic and nondiabetic persons, respectively (*P* = 0.0004) [85]. The advantages of PET over SPECT include the lower radiation patient exposure (due to the shorter physical half-lives of PET perfusion tracers) and more robust attenuation correction (leading to higher diagnostic accuracy in women and patients with larger body habitus) [86].

Stress echocardiography (either pharmacologic or with exercise) has similar diagnostic accuracy for CHD as stress testing with rMPI [87]. The diagnostic endpoint of exercise and pharmacological stress echocardiography is new or worsening wall motion abnormalities and changes in global LV function during or immediately after stress. In addition to the detection of inducible wall motion abnormalities, most stress echocardiography includes screening images to evaluate resting ventricular function and valvular abnormalities [50]. The presence and extent of resting LV dysfunction and ischaemia found with dobutamine or dipyridamole stress echocardiography are predictive of death, in both diabetic and nondiabetic individuals [58, 59]. Stress echocardiography is an observer- and patient-dependent procedure, the accuracy of which depends on the experience of the interpreter as well the acoustic windows available during stress testing. The use of intravenous ultrasound contrast agents can improve endocardial border delineation and can result in improved diagnostic accuracy [88].

Another modality that can be used for the evaluation of CHD is cardiac magnetic resonance (CMR) imaging, which provides an accurate means of assessing myocardial structure and function and enables characterization of the range of myocardial diseases from ischemic to inflammatory and various types of cardiomyopathy [89]. Delayed gadolinium enhancement CMR is able to directly visualize myocardial infarction in vivo [90]. The presence of late gadolinium hyperenhancement as a marker of prior MI in diabetic patients with unsuspected CHD has been linked with a 4-fold increased risk of major adverse cardiovascular events and a 7-fold increased risk of mortality [49].

Histological studies have shown that the extent of coronary artery calcium (CAC) is closely associated with total coronary artery atherosclerotic plaque burden [91]. Furthermore, CAC scores predict incident CHD in the general population [92] and patients affected by type 2 DM harbor larger amounts of CAC than nondiabetic patients of a similar age [93]. Additionally, the extent [94] and prevalence [95] of CAC in patients with type 2 DM asymptomatic for CHD is similar to that of patients with established CHD but without DM [96]. Interestingly, the extent of CAC has been shown to be associated with the prevalence of inducible ischaemia by SPECT-MPI [97]. Several studies [68, 98, 99], although not all [100], have demonstrated that increased CAC in persons with metabolic syndrome and/or diabetes is associated with increased prevalence of myocardial ischaemia, cardiac events, and mortality. In nondiabetic persons, the CAC score threshold at which the prevalence of ischaemia increases substantially is >400 Agatston units [97], although in diabetic patients this threshold has been reported to be lower [93]. Furthermore, sequential CAC imaging has been implemented as a means to assess atherosclerosis progression and progression of CAC has been shown to be a strong predictor of future MI [101]. On the other hand, a high proportion of adults with diabetes have zero or a very low CAC score (<10 Agatston units) [99, 102] but a CAC score of zero does not completely exclude CHD (in other words, CAC scoring does not allow differentiation between obstructive and nonobstructive CHD) [103]. In summary, since CAC measurement provides strong risk stratification of patients with diabetes, with an increase in mortality for each increase in CAC score category, is less expensive than SPECT/MPI, and has less radiation exposure, the overall evidence supports the class IIb indication in the 2013 ACC/AHA guidelines, claiming that the use of CAC scanning “may be appropriate” for risk stratification and guiding management in the asymptomatic DM patient [104].

Noninvasive coronary angiography can be performed with either multidetector-row CT (MDCT) [105] or magnetic resonance imaging (MRI) techniques, with better sensitivity and specificity for the MDCT compared to cardiac MRI [106]. For patients in whom CHD screening is being performed, CAC scoring and coronary CT angiography can directly identify the presence of atherosclerotic CHD, although neither test is able to provide functional information (i.e., impaired blood flow resulting in ischaemia) [107]. Any benefit of these newer noninvasive CHD screening methods, such as computed tomography and computed tomography angiography, to identify patient subgroups for different treatment strategies remains unproven and is thus not currently routinely recommended for using [108].

## 3. Safety of Screening Tests

It is reasonable and ethical to accept a certain risk for diagnostic tests applied to sick patients when they seek medical advice for specific complaints. However, it is quite another matter to subject presumably healthy people to risks. In such circumstances, the procedure should be particularly safe. This is partly because the chances of finding disease in healthy people are generally low. Thus, concerns have been raised about possible long-term risks with the increasing use of CT scans to screen for CHD. The radiation dose of CT scans varies by type, with a CT scan for coronary calcium on average being the equivalent of about 30 chest X-rays. One estimate of risk projected 29,000 excess cancers as a result of 70 million CT scans performed for various reasons in the United States in 2007 [109]. If these concerns are correct, CT scans used to screen for early CHD in asymptomatic individuals could themselves cause cancer over subsequent decades.

Adverse effects of screening tests include discomfort during the test procedure, risks related to the screening test per se (e.g., allergic reactions, long-term radiation effects, or perforation of coronary vessels during coronary arteriography), false-positive test results (with resulting needless workups and negative labeling effects [inconvenience and expense in obtaining follow-up procedures]), and overdiagnosis.

## 4. Studies of Screening for Asymptomatic CHD in Diabetes

As already stated, the primary purpose of screening for CHD in PWD would be to identify persons whose prognosis could be improved with an intervention (in this case, medical therapy for risk factors or coronary revascularization). In nearly all persons with diabetes, the results of screening will generally not change pharmaceutical medical therapy, since aggressive preventive measures, such as control of blood pressure and lipids, would already be indicated. Only in the case of aspirin administration, since there are real risks of gastrointestinal bleeding [110], one could argue that detection of CHD by screening might justify the therapy and might alter the risk/benefit ratio in favor of aspirin use [111]. The same applies to intensification of antilipid therapy [112] in these patients.

In observational studies, there is a well-defined relationship between the extent and severity of myocardial ischaemia and the rate of occurrence of major CHD events [113]. Despite this observation, however, the role of interventional therapy in treating ischaemia (on top of GDOMT) is ill-defined, and consequently, the role of screening procedures needs to be carefully examined.

Screening for CHD should be distinguished from the estimation of risk for CHD. By definition, both are performed in asymptomatic persons, and both aim to improve outcomes with interventions, if indicated. However, screening for CHD identifies existing disease, while estimating the risk of CHD does not directly identify existing disease but rather the likelihood of any future event related to CHD. The most important issue, however, is the effect of screening on hard outcomes (morbidity and mortality). Several prospective randomized trials have evaluated the impact of routine screening for subclinical CHD and the effect of therapy on outcomes of asymptomatic patients with type 2 diabetes. Taken altogether, these studies have shown no significant improvement in outcomes among patients who underwent screening [44, 114]. Certainly, knowledge of presence of occult coronary atherosclerosis or significant CHD found by screening might lead to better compliance with medical therapy, but this has also not been definitively demonstrated [115].

An initial small study, conducted more than 10 years ago, had actually shown some benefit of screening for asymptomatic CHD in DM [116]. In that study, 141 asymptomatic persons with T2D, admitted to the hospital for uncontrolled hyperglycaemia, were randomized into a screening arm for CHD (with an exercise ECG test and dipyridamole stress echocardiography) or a control arm. If one screening test was abnormal, coronary angiography was performed, followed by revascularization (CABG or PCI) for stenoses > 50%. After a mean follow-up of 53.5 months, the proportion of all cardiac events in the screened arm was significantly lower (*P* = 0.018), but with no difference in mortality.

In the largest study conducted to date (Detection of Ischemia in Asymptomatic Diabetics (DIAD study)) [117], 1123 type 2 diabetic patients without CHD symptoms at baseline were randomized to receive an adenosine rMPI, compared to no screening. In the screened group, the overall prevalence of silent myocardial ischaemia was 22%. Of note, no guidance was given to the treating physicians regarding management of patients with ischaemia on rMPI. After a mean follow-up of 4.8 years, there was no significant difference in the primary endpoint (cardiac death or nonfatal MI) between the screening and no-screening groups (2.7% versus 3.0%, resp.). Of note, a small proportion of people underwent a coronary angiogram within 120 days after screening (only 4.4% of the screened population versus 0.5% of the unscreened, *P* < 0.01) and even less underwent revascularization (1.6% versus 0.6%, resp., *P* = 0.03).

In a similar, smaller study of 631 asymptomatic patients with T2D and at least two other CHD risk factors conducted in France, the DYNAMIT (Do You Need to Assess Myocardial Ischemia in Type-2 diabetes) investigators randomized patients to either screening with rMPI (with symptom-limited bicycle exercise or dipyridamole SPECT) or no screening [118]. In the screened group, the prevalence of silent myocardial ischaemia was 21.5%, similar to the DIAD study. The study was discontinued prematurely because of difficulties in recruitment and a lower-than-expected event rate. After a mean follow-up of 3.5 years, there was no significant difference in the composite primary endpoint (death from all causes, nonfatal MI, nonfatal stroke, or heart failure requiring emergency intervention) between the screening and the nonscreening group (2.6% versus 2.4% annually; adjusted HR: 1.0; 95% CI: 0.59–1.71).

Equally negative results were found in a subsequent large study examining the benefit of screening for CHD in 900 diabetic patients (type 1 or 2) without prior CVD (FACTOR-64 trial) [119]. In contrast to the DIAD and DYNAMIT trials in which screening of asymptomatic patients with T2D was based on the identification of significant myocardial ischaemia using a functional stress test, the FACTOR-64 trial, conducted in the United States, evaluated the extent and severity of coronary atherosclerosis using an anatomic test (coronary computed tomography angiography (CCTA)). Also, contrary to the previous studies, it provided specific treatment guidance to the physicians, based on the CT results. Among patients randomized to CCTA screening, the prevalence of mild, moderate, and severe CHD was 31%, 46%, and 12%, respectively. After a mean follow-up of 4 years, there was no significant difference in the primary endpoint (composite of all-cause mortality, nonfatal MI, or unstable angina) following screening with CCTA (6.2% versus 7.6% without screening; HR: 0.8; 95% CI: 0.5–1.3).

Screening and revascularization of silent CHD in diabetic patients also failed to demonstrate a significant reduction in cardiac events and heart failure (HF) episodes in the Italian DADDY-D trial (Does coronary Atherosclerosis Deserve to be Diagnosed earlY in Diabetic patients?), where 520 diabetic patients without known CHD were randomly assigned to undergo screening for silent myocardial ischaemia (with exercise treadmill test) followed by revascularization if needed or to continue follow-up [120]. The reduction of cardiac death or nonfatal MI represented the primary aim; the secondary aim was the prevention of HF. After a mean follow-up of 3.6 years, there was no difference in cardiac events (HR = 0.85, 95% CI: 0.39–1.83, *P* = 0.678) or the occurrence of first HF episode (HR = 0.27, 95% CI: 0.06–1.31, *P* = 0.083).

A meta-analysis of all these 5 trials [114] corroborated the negative value of screening for asymptomatic CHD in diabetes. With a total number of 3315 asymptomatic diabetic patients included, and after 117 all-cause deaths and 100 cardiac events, it was shown that screening for CHD was not associated with a decrease in the risk for all-cause mortality (RR: 0.95 [95% CI: 0.66 to 1.35]) or cardiac events (RR: 0.72 [95% CI: 0.49 to 1.06]). This nonsignificant trend towards fewer cardiac events favouring the screening group seems to be driven by the study of Faglia et al. [116], which was the smallest and oldest study included in the analysis, with seemingly the poorest quality of patient treatment (patients had an unfavourable clinical profile, represented by the worst glycaemic control, the highest blood pressure, the greatest prevalence of smoking, and the lowest use of statins and aspirin in comparison with the other studies).

Complementary to the previous trials, the BARDOT trial (Basel Asymptomatic high-Risk Diabetics' Outcome Trial) evaluated the prognostic implications of medical versus invasive treatment in asymptomatic patients with T2D and abnormal screening test results (MPI-SPECT) [121]. In this study of 400 asymptomatic patients with T2D at high risk for CHD conducted in Switzerland and Germany, all patients underwent stress rMPI, which identified silent ischaemia in 88 participants (22%), similar to the DIAD and DYNAMIT trials. These patients with abnormal stress rMPI were then randomized to medical therapy alone versus medical therapy plus invasive coronary revascularization (with PCI and stent placement or CABG). The primary outcome was a combination of major adverse cardiac events (MACE: cardiac death, MI, and symptom-driven revascularization) and worsening rMPI findings at 2-years follow-up. Patients with abnormal MPS randomized to medical versus invasive-medical strategies had similar hard event rates ((HR: 0.36; 95% CI: 0.07 to 1.81; *P* = 0.215), but more ischemic or new scar findings on repeat scintigraphy (54.3% versus 15.8%; *P* < 0.001), implying that this kind of intervention could possibly ultimately reduce the risk of new downstream complications if broadly applied in the population [122]. The BARDOT trial results also slightly challenged the findings of the DIAD study (which included very low-risk patients and showed that rMPI screening is not effective at all in asymptomatic DM persons [117]), since in BARDOT, ischaemia testing in patients with diabetes at high coronary risk separated patients with CAD progression from those with a more benign course (in patients with normal MPS at baseline, MACE occurred in 2.9% and ischemia or new scar in 3.2%, whereas patients with abnormal baseline MPS had more MACE [9.8%] and ischemia or new scar [34.2%] at 2 years follow-up), suggesting also that at least the subclinical progression to silent CHD may be reduced with invasive and medical treatment compared with medical management only.

Similar to these findings, the Impact of inDucible Ischemia by Stress MPS (IDIS) trial [123] showed that addition of MPS imaging data to a prediction model based on traditional risk factors and ECG stress test data significantly improved CHD risk classification in 822 high-risk diabetic patients. Overall, 301 patients were reclassified to a higher risk category, with an event rate of 28%, and 26 to a lower risk category, with an event rate of 15% (net reclassification improvement (NRI): 0.25, 95% confidence interval (CI): 0.15–0.34). Patients at the lowest baseline risk category (3% to <5% risk) achieved a substantially higher NRI than the overall cohort (53% were reclassified at higher risk and 25% at lower risk, NRI: 0.42, 95% CI: 0.07–0.76), and therefore, patients in this category appear to be those who would benefit the most from a strategy that includes MPS data. Since in IDIS trial many of the participants were symptomatic or had a prior MI, the conclusions cannot be readily applied to a lower risk group of asymptomatic patients, with a substantially lower pretest risk of significant CAD [115]. However, in a subgroup analysis of the IDIS data [124], 436 consecutive asymptomatic diabetic patients who underwent stress-rest gated MPS were investigated and 27% were found to have an abnormal MPS. At multivariable analysis, poststress left ventricular ejection fraction (LVEF) and stress MPS ischaemia were independent predictors of CHD death or MI (both *P* < 0.01). NRI by adding MPS results to a model including pretest CHD likelihood was 0.25 (95% CI: 0.06–0.44). Parametric survival analysis showed the highest probability of CAD death or MI and the major risk acceleration in time in patients with stress MPS ischaemia and poststress LVEF < 45%. Together with other observational studies and meta-analyses [125, 126] of asymptomatic diabetic patient populations, these data show that silent ischaemia can be detected with screening and that such ischaemic findings are associated with an increased risk of cardiac events. Of course, such observational studies do not prove that outcomes are any better than optimal medical therapy, particularly in lower risk asymptomatic patients, which is the really most important issue [127].

## 5. Efficacy of Interventions for Early-Detected CHD

Although silent myocardial ischaemia has been associated with an increase in cardiac event rates compared to those without evidence of ischaemia in older studies [128–130], recent studies suggest that, in the contemporary GDOMT era, the presence of ischaemia is not related to the risk of death or MI in patients with SIHD [131–133] (in the older trials, the use of secondary prevention medications was substantially lower than that in the recent strategy trials [134]). And most importantly, studies that looked at the benefit of an invasive approach (PCI or CABG) together with optimal medical treatment of patients with stable, asymptomatic CHD compared to optimal medical treatment only (COURAGE and BARI-2D studies) have failed to show any benefit for the invasive approach [135, 136].

Specifically, in the COURAGE (Clinical Outcomes Using Revascularization and Aggressive Drug Evaluation) trial, 2287 patients with stable CHD were randomly assigned to either aggressive medical therapy alone or aggressive medical therapy plus PCI with bare-metal stenting [135]. Patients were required to have both objective evidence of ischaemia and significant disease in at least one coronary artery. Actually, 87% of participants were symptomatic, and only 34% had diabetes, so the study does not apply exclusively to silent CHD in diabetes. All patients received optimal medical therapy as indicated (ACE inhibitors or ARBs, statins, other lipid-lowering medications, aspirin, beta-blockers, calcium channel blockers, and nitrates). During a median follow-up of 4.6 years, there was no significant difference between the two treatment strategies for the primary end point of death from any cause and nonfatal MI (cumulative incidence approximately 19% in both groups; HR: 1.05; 95% CI: 0.87–1.27; *P* = 0.62). In addition, there was no significant difference in the rates of hospitalization for acute coronary syndrome (approximately 12% in both groups; HR: 1.07; 95% CI: 0.84–1.37; *P* = 0.56). Patients in the PCI group, however, underwent significantly fewer subsequent revascularization procedures (21% versus 33%, HR: 0.60, 95% CI: 0.51–0.71). In a subsequent report comprising 1121 participants of the initial study at 15 years of follow-up (median 6.2 years), again no significant difference in the rate of death was found in the two groups (24% and 25%, resp., adjusted HR: 1.03; 95% CI: 0.83–1.21; *P* = 0.76) [137].

Following the COURAGE trial, the results from the National Institutes of Health-National Heart, Lung and Blood Institute- (NIH-NHLBI-) sponsored BARI 2D (Bypass Angioplasty Revascularization Investigation 2 Diabetes) study were reported [136]. In that study, 2368 patients with T2D and stable ischaemic CHD were enrolled. Ischaemic CHD was defined as either a ≥50% stenosis of a major epicardial coronary artery associated with a positive stress test or ≥70% stenosis and classic angina. Prior to randomization to either revascularization (either PCI or CABG surgery) with intensive medical therapy (IMT) within four weeks or to IMT alone, patients were allocated in either the CABG or PCI stratum, as determined a priori by the responsible physician to be the most appropriate therapy for each patient. At 5 years, the primary end points of the rates of survival or freedom from major cardiovascular events (death, MI, or stroke) did not differ significantly between the revascularization group and the IMT alone group (88.3% versus 87.8% and 77.2% versus 75.9%, resp.). However, in subgroup analysis, the rate of freedom from major cardiovascular events was significantly higher in the CABG plus IMT stratum compared to the corresponding IMT stratum (77.6% versus 69.5%), predominantly attributable to a reduction in nonfatal MI (10.0% versus 17.6%; *P* = 0.003) [138]. The rates for this end point were not significantly different between the PCI stratum and the corresponding IMT group (77.0% versus 78.9%, resp.). The lower event rate in the CABG plus IMT stratum was attributed to a preference of the treating physicians for CABG, rather than PCI, in patients with more extensive disease (including more triple-vessel and proximal left anterior descending coronary artery disease) [139]. Thus, this finding that CABG might be better than medical therapy alone for preventing major CVD events in diabetes must be interpreted with caution, as the allocation to PCI or CABG was not randomized.

Furthermore, studies using fractional flow reserve (FFR) to guide decision making have not yielded firm conclusions yet. FFR is a pressure wire-based index that is used during coronary angiography to assess the potential of a coronary stenosis to induce myocardial ischaemia [140]. The aim of the FAME-2 trial was to determine whether FFR-guided PCI with drug-eluting stents plus the best available medical therapy is superior to the best available medical therapy alone in reducing the rate of death, myocardial infarction, or unplanned hospitalization leading to urgent revascularization among 888 patients with stable CHD and FFR < 0.8 [141]. There was no significant difference in mortality (HR: 0.33, CI: 0.03–3.17, *P* = 0.31) or MI (HR: 1.05, CI: 0.51–2.19, *P* = 0.89), partially because the trial's data safety monitoring board recommended enrollment to be stopped prematurely, after an interim analysis revealed a highly statistically significant decrease in unplanned hospitalization leading to urgent revascularization in the PCI arm. This drove a significant reduction in the composite primary endpoint (death, MI, or hospitalization for urgent revascularization) for FFR-guided PCI plus medical therapy as compared to medical therapy alone (HR: 0.32, 95% CI: 0.19–0.53, *P* < 0.0001). Thus, firm conclusions from this trial regarding the role of invasive procedures on hard end-points of CHD morbidity and mortality cannot be drawn.

Apart from that, meta-analyses of SIHD strategy trials do not support a difference in prognosis between routine revascularization and GDOMT only [142–144]. There is also some evidence that silent myocardial ischaemia may reverse over time with intensification of medical therapy [145].

Cost-effectiveness analyses also have not favoured interventional procedures in the randomized trials so far [146, 147].

## 6. Recommendations of Major Scientific Organizations

Based on the above-mentioned data, the American Diabetes Association does not recommend screening of asymptomatic diabetic patients with high atherosclerotic CVD risk [148], in part because these high-risk patients should already be receiving intensive medical therapy, an approach that provides similar benefit as invasive revascularization. They recommend investigation for CHD in the presence of any of the following: atypical cardiac symptoms (i.e., unexplained dyspnea, chest discomfort); signs or symptoms of associated vascular disease including carotid bruits, transient ischaemic attack, stroke, claudication, or peripheral arterial disease; or ECG abnormalities (e.g., Q waves) [108].

The European Society of Cardiology (ESC)/European Association for the Study of Diabetes (EASD) in their 2013 Guidelines on diabetes, prediabetes, and CVD concludes that in asymptomatic patients routine screening is controversial and still under debate [149]. In addition, they highlight the need for better definition of the characteristics of the patients who should be screened for CHD, stating that screening for silent myocardial ischaemia may be considered in selected high-risk patients with diabetes, such as patients with peripheral artery disease or high CAC score or with proteinuria.

While recommendations of other major scientific organizations vary regarding the optimal approach to screening for CHD, no professional society guideline or consensus statement advocates for universal screening.

The United States Preventive Services Task Force (USPSTF) recommends against routine screening in adults at low risk for CHD events and also concludes that there is insufficient evidence to recommend for or against routine screening in adults at increased risk for CHD events [150].

The American College of Cardiology/American Heart Association (ACC/AHA) guidelines for exercise testing also issued a similar recommendation in 2002, that there is little evidence to support routine exercise testing in asymptomatic adults [151]. They further concluded that the weight of evidence favours evaluation of asymptomatic patients with diabetes who plan to begin a vigorous exercise programme and that exercise testing can be considered (although the weight of evidence is less clear) in the following patient populations: patients with multiple risk factors for CHD as a guide to risk reduction therapy, men over age 45 years and women over age 55 years who are presently sedentary and plan to start a vigorous exercise programme, and patients who are involved in occupations linked to public safety. Exercise testing can also be considered in patients who have undergone electron beam computed tomography (EBCT) and have a coronary calcium score above the 75th percentile.

The American College of Physicians (ACP) recommends against screening low-risk, asymptomatic adults with resting ECG, stress ECG, stress echocardiography, or stress myocardial perfusion imaging [152].

## 7. Unresolved Issues and Future Studies

Although the results from recent stable ischaemic heart disease randomized clinical trials have been negative as to whether the addition of coronary revascularization to GDOMT reduces death or major CVD events [135, 136], there are still some unresolved and confusing issues around this matter. For example, revascularization (PCI or CABG) compared with GDOMT was associated with reduced death and MI among 9676 propensity-matched “real-world” patients meeting COURAGE eligibility criteria [153]. Furthermore, in some observational studies, a strong relationship between the extent of ischaemia and subsequent death and/or MI and a possible benefit from revascularization has been observed. When at least moderate ischaemia (>10%) was present, patients undergoing revascularization had fewer cardiac deaths than patients who were not revascularized [154]. Adding to the confusion, in the COURAGE nuclear substudy [155], among 105 patients with >10% ischaemia who had follow-up scans 1 year later, those who received PCI were more likely to experience significant ischaemia reduction than GDOMT alone (78% versus 52%; *P* = 0.007). Compared to those with persistent or worsening ischaemia, patients with ischaemia reduction by whatever means (i.e., PCI or GDOMT) had lower unadjusted risk for death or MI. However, a subsequent COURAGE analysis of outcomes by treatment group in 468 patients with at least moderate ischaemia on baseline rMPI showed no reduction in death or MI from the addition of PCI to GDOMT [156]. At the same time, though, crossover to PCI for progressive symptoms or ACS was required in 32% of GDOMT patients during a median 4.6-year follow-up in COURAGE and this reduces power to demonstrate differences.

Although the image of coronary arteries as kitchen pipes clogged with fat is simple, familiar, and evocative, it is also wrong [157]. The truth is that the angiogram is a poor discriminator of physiological lesion significance. Many lesions that appear angiographically severe may not produce ischaemia, and conversely, ischaemia may be present despite a benign angiographic lesion [158, 159]. It may be that revascularization in SIHD may not be beneficial because not all anatomically obstructive coronary stenoses produce ischaemia, or because not all high-grade coronary stenoses result in cardiac death and/or MI, or conversely, because most cases of cardiac death and/or MI arise from angiographically mild coronary lesions, which are not revascularized [160].

In addition, the studies mentioned were performed over several decades, and controlling for evolution in general medical practice is not possible. Indeed, many of these studies are of questionable relevance to contemporary practice today, given advances in GDOMT and revascularization techniques and devices. Of note, bare-metal stents (BMS) were used in most PCI versus GDOMT trials to date (including the COURAGE and BARI 2D studies). First-generation drug-eluting stents (DES) markedly reduce recurrent ischaemia compared with BMS [161], resulting in fewer hospitalizations for repeat revascularization [162]. Compared with BMS and first-generation DES, second-generation DES may further reduce death and MI and enhance event-free survival [163].

A potential explanation for failure of revascularization to reduce the incidence of death or MI in prior SIHD strategy trials is that lower risk patients were permitted into these trials, diluting the power to show a benefit from revascularization. Since it has been hypothesized that there is a level of ischaemia above which a revascularization strategy might result in benefit regarding cardiovascular events, this has mandated the performance of a specific study to determine the optimal approach to managing patients with SIHD, with moderate-to-severe ischaemia, and symptoms that can be controlled medically. The ongoing ISCHEMIA trial (International Study of Comparative Health Effectiveness With Medical and Invasive Approaches) (NCT01471522) is an NHLBI-funded international randomized controlled trial that began in 2012, with a primary aim of recruiting 8000 participants in order to determine whether an initial invasive strategy of cardiac catheterization and optimal revascularization (with PCI or CABG, as determined by the local heart team) plus GDOMT will reduce the primary composite endpoint of cardiovascular death or nonfatal MI in SIHD patients with moderate or severe ischaemia and medically controllable or absent symptoms, as compared with an initial conservative strategy of GDOMT alone, with catheterization reserved for failure of GDOMT. The major secondary endpoint is the angina-related quality of life. Other important secondary endpoints are health resource utilization, costs, and cost-effectiveness. In addition to the main trial, 1000 additional patients with advanced chronic kidney disease (estimated glomerular filtration rate < 30 ml/min or on dialysis) will be randomized in a parallel NHLBI-funded ISCHEMIA-CKD ancillary substudy. Blinded CCTA is performed before randomization in participants with normal renal function to exclude those with significant left main artery disease and no obstructive CHD. This is in contrast to COURAGE and BARI 2D trials, where enrollment was not predicated on core laboratory confirmation of any significant degree of ischaemia. The rationale for including multiple imaging modalities is to enhance the generalizability of findings to the diverse modalities that are available to practicing clinicians caring for SIHD patients around the globe.

The ISCHEMIA study thus aims to address limitations of previous strategy trials by (1) enrolling patients before catheterization, so that anatomically high-risk patients are not excluded; (2) enrolling a higher-risk group with at least moderate ischaemia; (3) minimizing crossovers; (4) using contemporary DES and physiologically guided decision making (FFR) to achieve complete ischaemic (rather than anatomic) revascularization; and (5) being adequately powered to demonstrate whether routine revascularization reduces cardiovascular death or nonfatal MI in patients with SIHD and at least moderate ischaemia [45].

## 8. Conclusions

Diabetes is well known to significantly increase CVD risk, but cannot be considered a CHD equivalent, due to great heterogeneity of the patients. Nevertheless, life-time risk of CHD seems to be quite high in almost all people with the disease, which calls for individualized approach and evaluation for the presence and possible treatment of a great variety of other frequently coexisting risk factors that can increase this risk.

Apart from risk factor treatment, however, the value of invasive treatment of coronary atherosclerosis (except for the case of acute coronary syndromes) remains unsettled, because all prior randomized trials have limitations and are pointing towards equipoise [44, 45], and thus, routine screening for silent CHD in asymptomatic persons with DM is not currently recommended, as long as cardiovascular risk factors are treated (Table 2) [108]. It is hoped that the ongoing ISCHEMIA trial will give more definitive answers to the current uncertainties pertaining proper treatment of SIHD.

## Figures and Tables

**Table 1:** Screening methods for detecting asymptomatic coronary artery ischaemia in patients with diabetes.

Screening methods	Detection of prevalent CHD	Comments
*Functional tests*		
Resting electrocardiogram (ECG)	Low sensitivity and specificity	Widely available, very low cost
Exercise ECG	Moderate sensitivity (45–61%) and specificity (70–90%)	Relatively low cost, widely available Many patients unable to exercise Some have uninterpretable baseline ECGs
Radionuclide single proton emission computed tomography (SPECT) myocardial perfusion imaging (MPI)	Good sensitivity (80–90%) and specificity (75–90%) The most widely used test to assess silent myocardial ischaemia	Moderate to high cost Widely available High negative predictive value (95%) Image quality affected by body habitus and large breasts Screening of asymptomatic patients not prognostically useful unless high-risk patients are selected
Myocardial perfusion imaging (MPI) with positron emission tomography (PET)	High sensitivity for myocardial viability studies Accurate global and regional measurements of myocardial perfusion, blood flow, and function at stress and rest in a single study	Better image quality because of higher spatial resolution, less scattered, and fewer attenuation artifacts Lower radiation exposure than SPECT Costly, not universally available
Stress echocardiography (i) Exercise stress echo (ii) Pharmacologic stress echo (dobutamine, adenosine, and dipyridamole)	The sensitivity and specificity are satisfactory (80–85%) Able to assess LV function and valvular abnormalities	Low cost, widely available Operator dependent Difficulty in interpreting the images in obese persons

*Anatomic (imaging) techniques*		
Coronary artery calcium score (CAC)	CAC more prevalent in people with diabetes than nondiabetes Closely associated with total coronary artery atherosclerotic plaque burden Predicts incident ischaemia, CHD morbidity and mortality	Moderate to high cost No differentiation between obstructive and nonobstructive CHD Up to 25% of patients have minimal or no CAC at the time of screening
Multidetector-row computed tomography (MRCT) angiography	High sensitivity (83–99%) and specificity (93–98%)	Good sensitivity, specificity, and negative predictive value. High radiation doses High cost
Magnetic resonance imaging (MRI)	Good sensitivity (83–90%) and specificity (72–84%) Delayed gadolinium hyperenhancement linked to increased risk of major cardiovascular events Not adequately investigated	Able to assess myocardial structure and function and characterize ischemic, inflammatory and various types of cardiomyopathies High cost

**(a):** 

Silent CHD/ischaemia screening studies									
Study name	Screening method	Patients (*n*)	Age (years)	Smoking (%)	Statin use (%)	Aspirin use (%)	Mean follow-up (years)	Silent CHD-ischaemia (%)	Main outcome
Faglia et al. ([116])	Exercise ECG and dipyridamole-stress echo	71	58.7 ± 8.3	46	28	9	4.4	21.4	In the screened arm, the proportion of all events (*P* = 0.018) as well as the proportion of major to minor events (*P* = 0.006) was significantly less
	No screening	70	61.5±8.1	55	21	12		NA	
DIAD (Young et al. [117])	Stress scintigraphy	561	60.7 ± 6.7	10	37	43	4.8	22	No difference in cardiac death or nonfatal MI (HR): 0.88; 95% CI: 0.44–1.88; *P* = 0.73
	No screening	562	60.8 ± 6.4	9	41	46		NA	
DYNAMIT (Lièvre et al. [118])	Bicycle exercise test or stress scintigraphy	316	64.1 ± 6.4	17	33	39	3.5	21.5	No difference in composite primary endpoint (death from all causes, nonfatal MI, nonfatal stroke, or heart failure requiring emergency intervention) between the screening and the nonscreening group (2.6% versus 2.4% annually; adjusted HR: 1.0; 95% CI: 0.59–1.71)
	No screening	315	63.7 ± 6.4	14	36	24		NA	
FACTOR-64 (Muhlestein et al. [119])	Coronary CT angiogram (CCTA)	452	61.5 ± 7.9	16	76	43	4.0	69	The primary outcome event rates not significantly different between the CCTA and the control groups (6.2% versus 7.6%; hazard ratio: 0.80 [95% CI: 0.49–1.32]; *P* = 0.38)
	No screening	448	61.6 ± 8.3	15	72	40		NA	
DADDY-D (Turrini et al. [120])	Exercise ECG	262	61.9 ± 4.8	40	39	29	3.6	7.6	No difference in cardiac events (HR = 0.85, 95% CI: 0.39–1.83, *P* = 0.678) or occurrence of first HF episode (HR = 0.27, 95% CI: 0.06–1.31, *P* = 0.083)
	No screening	258	62 ± 5.1	37	44	25		NA	

**(b):** 

Outcome studies									
Study name	Group randomization	Patients (*n*)	Age (years)	Smoking (%)	Statin use (%)	Aspirin use (%)	Mean follow-up(years)	Silent CHD-ischaemia (%)	Main Outcome
COURAGE (Boden et al. [135])	Medical therapy plus PCI with bare-metal stenting	1149	61.5 ± 10.1	23	86	96	4.6	NA (all participants had known CHD)	No difference for the primary endpoint of death from any cause and nonfatal MI (cumulative incidence approximately 19% in both groups; HR: 1.05; 95% CI: 0.87–1.27; *P* = 0.62). No significant difference in rates of hospitalization for acute coronary syndrome (approximately 12% in both groups; HR: 1.07; 95% CI: 0.84–1.37; *P* = 0.56). Patients in PCI group underwent significantly fewer subsequent revascularization procedures (21% versus 33%, HR: 0.60, 95% CI: 0.51–0.71)
	Medical therapy alone	1138	61.8 ± 9.7	23	89	95			
BARI 2D (Mori Brooks et al. [136])	Revascularization (PCI or CABG) with intensive medical therapy (IMT)	953	62.3 ± 8.8	10.4	94.6	93.5	5	NA (all participants had known CHD)	No difference in primary endpoints of survival or freedom from major CVD events (death, MI, or stroke) between the revascularization and IMT groups (88.3% versus 87.8% and 77.2% versus 75.9%, resp.)
	IMT alone	991	62.4 ± 9.0	11.2	95.4	94.2			
BARDOT (Zellweger et al. [121])	Positive MPI with SPECT (MPS)	87	65 ± 7	32	66	63	2	22	Patients with abnormal MPS randomized to medical versus invasive-medical strategies had similar hard event rates ((HR: 0.36; 95% CI: 0.07 to 1.81; *P* = 0.215), but more ischemic or new scar findings on repeat scintigraphy (54.3% versus 15.8%; *P* < 0.001)
	Negative MPS	313	63 ± 8	18	55	50			

CHD: coronary heart disease; ECG: electrocardiogram; echo: echocardiography; NA: not applicable; DIAD: Detection of Ischemia in Asymptomatic Diabetics; DYNAMIT: Do You Need to Assess Myocardial Ischemia in Type-2 diabetes; DADDY-D: Does coronary Atherosclerosis Deserve to be Diagnosed earlY in Diabetic patients; COURAGE: Clinical Outcomes Using Revascularization and Aggressive Drug Evaluation; BARI 2D: Bypass Angioplasty Revascularization Investigation 2 Diabetes; BARDOT: Basel Asymptomatic high-Risk Diabetics' Outcome Trial.
